# Early Induction of Human Regulatory Dermal Antigen Presenting Cells by Skin-Penetrating *Schistosoma Mansoni* Cercariae

**DOI:** 10.3389/fimmu.2018.02510

**Published:** 2018-10-31

**Authors:** Béatrice M. F. Winkel, Mirjam R. Dalenberg, Clarize M. de Korne, Carola Feijt, Marijke C. C. Langenberg, Leonard Pelgrom, Munisha S. Ganesh, Maria Yazdanbakhsh, Hermelijn Helene Smits, Esther C. de Jong, Bart Everts, Fijs W. B. van Leeuwen, Cornelis H. Hokke, Meta Roestenberg

**Affiliations:** ^1^Department of Parasitology, Leiden University Medical Center, Leiden, Netherlands; ^2^Interventional Molecular Imaging Laboratory, Department of Radiology, Leiden University Medical Center, Leiden, Netherlands; ^3^Department of Cell Biology and Histology, Academic Medical Center, Amsterdam, Netherlands; ^4^Department of Infectious Diseases, Leiden University Medical Center, Leiden, Netherlands

**Keywords:** schistosome, *Schistosoma mansoni*, cercaria, bilharzia, human, skin, macrophage, PD-L1

## Abstract

Following initial invasion of *Schistosoma mansoni* cercariae, schistosomula reside in the skin for several days during which they can interact with the dermal immune system. While murine experiments have indicated that exposure to radiation-attenuated (RA) cercariae can generate protective immunity which is initiated in the skin stage, contrasting non-attenuated cercariae, such data is missing for the human model. Since murine skin does not form a reliable marker for immune responses in human skin, we used human skin explants to study the interaction with non-attenuated and RA cercariae with dermal innate antigen presenting cells (APCs) and the subsequent immunological responses. We exposed human skin explants to cercariae and visualized their invasion in real time (initial 30 min) using novel imaging technologies. Subsequently, we studied dermal immune responses and found an enhanced production of regulatory cytokine interleukin (IL)-10, pro-inflammatory cytokine IL-6 and macrophage inflammatory protein (MIP)-1α within 3 days of exposure. Analysis of dermal dendritic cells (DDCs) for their phenotype revealed an increased expression of immune modulators programmed death ligand (PD-L) 1 and 2, and increased IL-10 production. *Ex vivo* primed DDCs suppress Th1 polarization of naïve T-cells and increase T-cell IL-10 production, indicating their regulatory potential. These immune responses were absent or decreased after exposure to RA parasites. Using transwells, we show that direct contact between APCs and cercariae is required to induce their regulatory phenotype. To the best of our knowledge this is the first study that attempts to provide insight in the human dermal *S. mansoni* cercariae invasion and subsequent immune responses comparing non-attenuated with RA parasites. We reveal that cercariae induce a predominantly regulatory immune response whereas RA cercariae fail to achieve this. This initial understanding of the dermal immune suppressive capacity of *S. mansoni* cercariae in humans provides a first step toward the development of an effective schistosome vaccine.

## Introduction

After penetrating the skin, larvae of the *Schistosoma mansoni* (*S. mansoni*) parasite, termed cercariae, transform into schistosomula and reside locally for several days, during which they penetrate the epidermal-dermal junction and eventually continue onward migration (1–3). Until now, the dynamic aspects of this invasion into human skin remain largely unknown. As the skin is an immune-competent organ containing various immune cells (4), it provides the first opportunity for host immune cells to recognize parasite antigens. This interaction could be important as it can drive an adaptive immune response against *S. mansoni* (5). Although it is widely accepted that schistosomes are able to direct immune responses via egg-induced immune modulation at late stages of infection, the modulatory effects during the initial stages are less well-defined.

Although human dermal immune responses to *S. mansoni* have not been studied to date, mouse models reveal a mixed immune response to cercariae. In mice, *S. mansoni* invasion induces inflammation, shown by a dermal infiltrate, which peaks by day 4 post infection (6, 7). From the reports on acute schistosomiasis syndromes it is clear that there is considerable inter-individual variability in the human immune responses to schistosome infection, reflected by variation in cercarial dermatitis and onset of Katayama fever (8–10). Analysis of murine dermal immune responses to *S. mansoni* larvae revealed an enhanced migration of innate antigen presenting cells (APCs) of such as macrophages (Mϕ) and dendritic cells (DCs), to the skin draining lymph node as well as an increase in their activation markers, MCH class II and CD86 (5, 7, 11–13). Nonetheless, exposure to cercariae does not readily induce protective immunity. This may be due to counteracting regulatory cytokine responses in the form of IL-10 and IL-1ra which are mounted in the dermis within 2 days post infection (7, 11, 14). Together these early innate responses in the dermis culminate in a short-lived mixed Th1/Th2 cytokine response in the skin draining lymph node which rapidly declines to baseline (7, 15) resulting in a failure to induce protective immunity against a subsequent infection. One possible way by which *S. mansoni* cercariae are suggested to achieve immune regulation is by the production of excretory/secretory (ES) products upon transformation into schistosomula, which can suppress (dermal) immune responses (7, 11, 12, 16–20). Proteomic analysis of skin invasion identified a variety of secreted enzymes and factors that are able to degrade host immune defense molecules (20).

APCs orchestrate the adaptive immune response to antigens and one molecular mechanism by which APCs are able to inhibit an adaptive immune response is the PD-1/PD-L1 (Programmed Death-1/Programmed Death Ligand-1) interaction. PD-L1 has been described as a regulatory marker on APCs and is linked to the induction of immunological tolerance (21–23). In tumor immunology, PD-L1 up regulation leads to immune-escape and T-cell anergy upon ligation with PD-1 (24–26), and PD-L1 has been shown to play a pivotal role in the polarization of naïve CD4^+^ T cells to regulatory T cells (Tregs) (27). The role of PD-L2, the other known PD-1 ligand, is less clear. In addition to cancer cells, many pathogens have been shown to exploit the PD-1 pathway in order to escape the host's immune response (26, 28–31). We aimed to determine whether this immune regulation pathway could potentially play a role in *S. mansoni* infection.

In contrast to non-attenuated cercariae, repeated exposure to radiation-attenuated (RA) cercariae induces protective immunity in animal models. RA cercariae yield a sustained IL12p40 mediated protective Th1 response, which has the capacity to kill migrating parasites in the host lungs (7, 32–37). It has been reasoned that delayed dermal migration of RA cercariae, and thus prolonged antigen exposure, leads to an enhanced and sustained pro-inflammatory dermal cytokine response (5, 38). Interestingly, in contrast to their non-attenuated counterparts, RA cercariae either induced delayed IL-10 responses (7) or failed to induce IL-10 at all (18). Corroborating this, IL-10 deficient mice mounted higher protective immunity after vaccination with RA cercariae (39). They also showed massive accumulation of inflammatory cells around invaded parasites, and a delay in schistosomula migration (18). Taken together, these findings illustrate the importance of IL-10 in the down-regulation of the dermal immune response used by *S. mansoni* for the continuation of the parasite life cycle in mice.

Despite the numerous studies of the interaction between *S. mansoni* larvae and murine skin, human dermal APC responses in *ex vivo* skin have not been studied. There are, however, substantial differences between murine and human skin; human skin is anatomically [thickness, muscle layers, dermal papillae, and hair follicle density (40, 41)], as well as immunologically distinct from murine skin and consequentially has different immune cell subsets (42) and differential expression of receptors on dermal APCs (43). These features may limit the clinical relevance of findings in mice and demand a set-up that enables immune studies in human skin.

**Figure 1:**
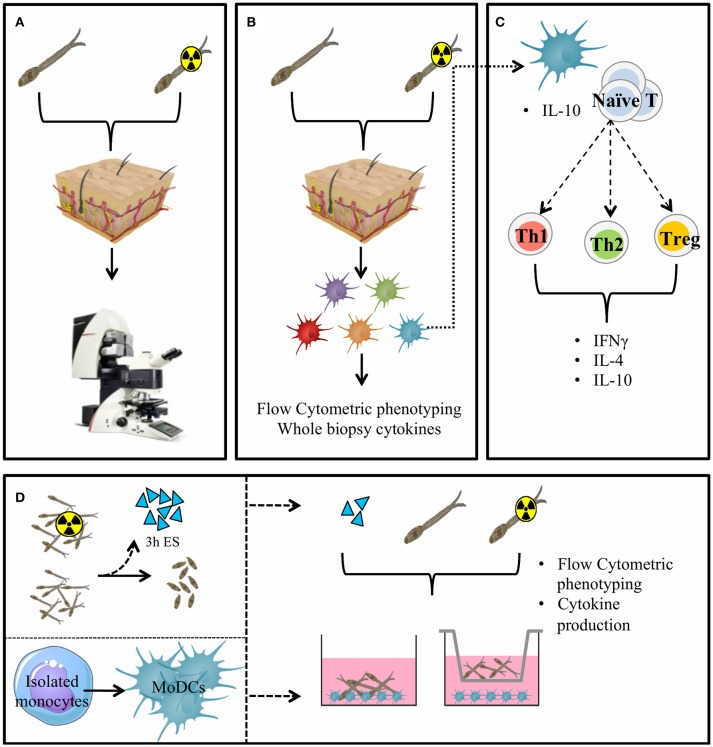
General presentation of the skin invasion set up and immunological markers studied. Skin invasion imaging setup: *S. mansoni* (RA) cercariae are irradiated or kept at RT. Human skin explant pieces are mounted on confocal microscopy dishes and cercariae are added to the epidermal side. Explants are imaged with a confocal microscope. **(A)** Skin explants are exposed to (RA) cercariae in water and cultured to harvest emigrating DDCs **(B)**. DDCs are co-cultured with naïve T cells to assess functional effects **(C)**. Monocyte derived dendritic cells **(D)** (MoDCs) are differentiated from human monocytes. Three hour ES products are generated from (RA) schistosomula. Stimulation of MoDCs with (RA) cercariae or their ES products in presence or absence of a transwell.

In this study we have used imaging technologies to monitor the human skin invasion of laboratory-reared *S. mansoni* (RA) cercariae *ex vivo*. We analyzed the ensuing human immune response characterizing the phenotype and PD-L1 expression on DDCs and their functional effects on CD4^+^ T cell polarization (Figure 1).

## Materials and methods

### Parasite materials

*S. mansoni* cercariae from the Puerto Rican-strain were shed from *Biomphalaria glabrata* watersnails for 2.5 h at 30°C in water. Radiation attenuation was performed by irradiating cercariae at room temperature to a total dose of 20krad using a Cesium radiation source.

For the production of cercarial excretory/secretory products (ES), (RA) cercariae were transformed into schistosomula *in vitro* by centrifugation of 100,000–300,000 cercariae at 1600 rpm for 5 min, after which supernatant was removed and replaced by 12.5 mL of pre-warmed (37°C) RPMI (Invitrogen, Carlsbad, CA, USA) supplemented with Penicillin, Streptomycin, 20 μM pyruvate and 20 μM L-glutamine (Sigma-Aldrich, Zwijndrecht, The Netherlands). Cercariae were incubated at 37°C and 5% CO_2_ for 20 min to induce transformation. Tails were separated from schistosomula bodies using an orbital shaker and schistosomula were collected and cultured for 3h at 37°C and 5% CO_2_ in a 96-wells plate (4000 schistosomula/mL at 100 μl per well). After the culture period, supernatant containing cercarial ES was collected and pooled.

### Skin explants

Human skin explants were obtained from collaborating hospitals immediately after abdominal skin reduction surgery (ERB number B18.009, see ethics statement) and kept at 4°C until use (within 6 h). Subcutaneous fat was removed and the epidermal side thoroughly cleaned with 70% ethanol. Skin pieces were wrapped around an electrically heated pad and placed upon petri dishes filled with either 15 ml water or 15 ml water containing (RA) cercariae. Twelve thousand (RA) cercariae were allowed to penetrate a circular skin piece with a diameter of 5 cm (surface area 19.6 cm^2^) for 30 min, after which the sample was removed, cleaned and biopsied using 6 mm punch biopsies. Biopsies were rinsed in RPMI supplemented with 0.1% fetal calf serum (FCS, Bodinco, Alkmaar, The Netherlands) and transferred to a 48 wells plate containing 1 ml RPMI 10% FCS per well supplemented with 500 U/ml GM-CSF. Emigrated immune cells were collected from the supernatant after 3 days, washed, filtered and stained for Flow Cytometric analysis or irradiated to a total dose of 3000 rad and brought into culture for co-culture assays.

### Visualization of cercarial invasion of human skin explants

Small skin explant pieces (4 × 8 mm) were placed into a confocal dish (ø35mm; MatTek Corporation). To enable imaging, the cercariae were labeled with the fluorescent cyanine dye Cy5-methyl-methyl [500 nM; Interventional molecular imaging group LUMC, Leiden, The Netherlands (Winkel et al., submitted)]. The labeled cercariae were added to the epidermal side of the skin piece and invasion was imaged using the time-lapse function of the Leica TCS (true confocal scanning) SP8X WLL (white light laser) microscope (Leica Microsystems, Wetzlar, Germany; 10x objective). Cy5-methyl-methyl was excited at 633 nm and emission was collected between 650 and 700 nm. The UV-laser (excitation: 405 nm, emission: 420–470 nm) was used to visualize skin structures (epidermis, epidermal-dermal junction, dermis) based on its auto fluorescence. The cercariae invasion was analyzed using the Leica Application Suite X software (Leica Microsystems, Wetzlar, Germany).

### Flow cytometric analysis

Emigrated dermal dendritic cells (DDCs) were distinguished from other immune cells by their forward and side scatter properties, in addition to high expression of CD11c and HLA-DR. The different DDC subsets were determined using the expression of CD1a, CD14 and CD141: CD141^+^CD14^−^ (referred to as CD141^+^ DDCs), CD1a^high^CD14^−^ (referred to as LCs), CD1a^int^CD14^−^ (referred to as CD1a+ DDCs) and CD1a^−^CD14^+^ (referred to as CD14^+^ DDCs). Antibodies used were HLA-DR-PerCP-ef710 (L243, eBioscience), CD11c-PE-Cy7 (B-Ly6, BD Pharmingen), CD80-BV650 (L307.4, BD Biosciences), CD1a-AF700 (HI149), CD141-BV421 (M80), lineage cocktail-APC (CD3/19/20/56; Biolegend), CD14-PE-Texas Red (Tuk4, Life Technologies), PDL1-APC (MIH1) and PDL2-PE or PECy7 (MIH18; eBioscience). All conditions were incubated with CD16/32 Fc receptor inhibitor (eBioscience) and Aqua live/dead staining (Invitrogen). Samples were measured using a FACS canto-II (BD Bioscience Franklin Lakes, NJ, USA) and analyzed in FlowJo^TM^ (FlowJo LLC, Ashland, OR, USA).

### Naïve CD4^+^ T cell co-culture

For analysis of T cell polarization, 5 × 10^3^ emigrated DDCs were irradiated (3000 rad) and co-cultured with 2 × 10^4^ allogeneic naïve CD4^+^ T cells isolated from buffy coat (Sanquin, Amsterdam, The Netherlands). Co-cultures were performed in the presence of staphylococcal enterotoxin B (10 pg/ml). On days 6 and 8, recombinant human IL2 (10 U/ml; R&D Systems) was added and the T cells were expanded until day 11. Intracellular cytokine production was analyzed after polyclonal restimulation with 100 ng/ml phorbol myristate acetate (PMA) and 1 μg/ml ionomycin (Sigma Aldrich) for 6 h. Brefaldin A (10 μg/ml; Sigma Aldrich) was added for the last 4 h of restimulation. Cells were fixed in 3.7% paraformaldehyde (Sigma Aldrich), permeabilized with permeabilization buffer (eBioscience), stained with antibodies against IL-4 and IFNγ (BD bioscience) and analyzed with flow cytometry.

In addition, 10^5^ expanded CD4 T cells were restimulated with antibodies against CD3 and CD28 for 24 h in a 96-wells plate. Supernatants were harvested and analyzed for IL-10 secretion using standard ELISA (Sanquin, Amsterdam, The Netherlands).

### J558-CD40L co-culture

DDCs were co-cultured with a CD40L expressing J558 myeloma line at a 1:1 ratio in a round bottom 96-wells plate. After 24 h supernatants were harvested and kept at −20 until analyzed for cytokine production by standard ELISA.

### Monocyte derived dendritic cells (MoDCs)

Monocytes were isolated from venous whole blood from healthy volunteers and differentiated as described previously (44). On Day 5, MoDCs were harvested, counted and re-cultured at 35 × 10^4^ cells/well in a 24 wells plate and rested for 24 h. On day 6 the immature MoDCs were stimulated with *S. mansoni* cercariae or RA cercariae or their ES products (in water, 100 cercariae/well), water control (equivalent volume to cercarial stimulation), LPS (100 ng/ml) or medium. For transwell experiments, transwell inserts with 8 μm pore size were used (Costar, Corning NY, USA).

## Results

### Invasion of cercariae into human skin explants

**Figure 2:**
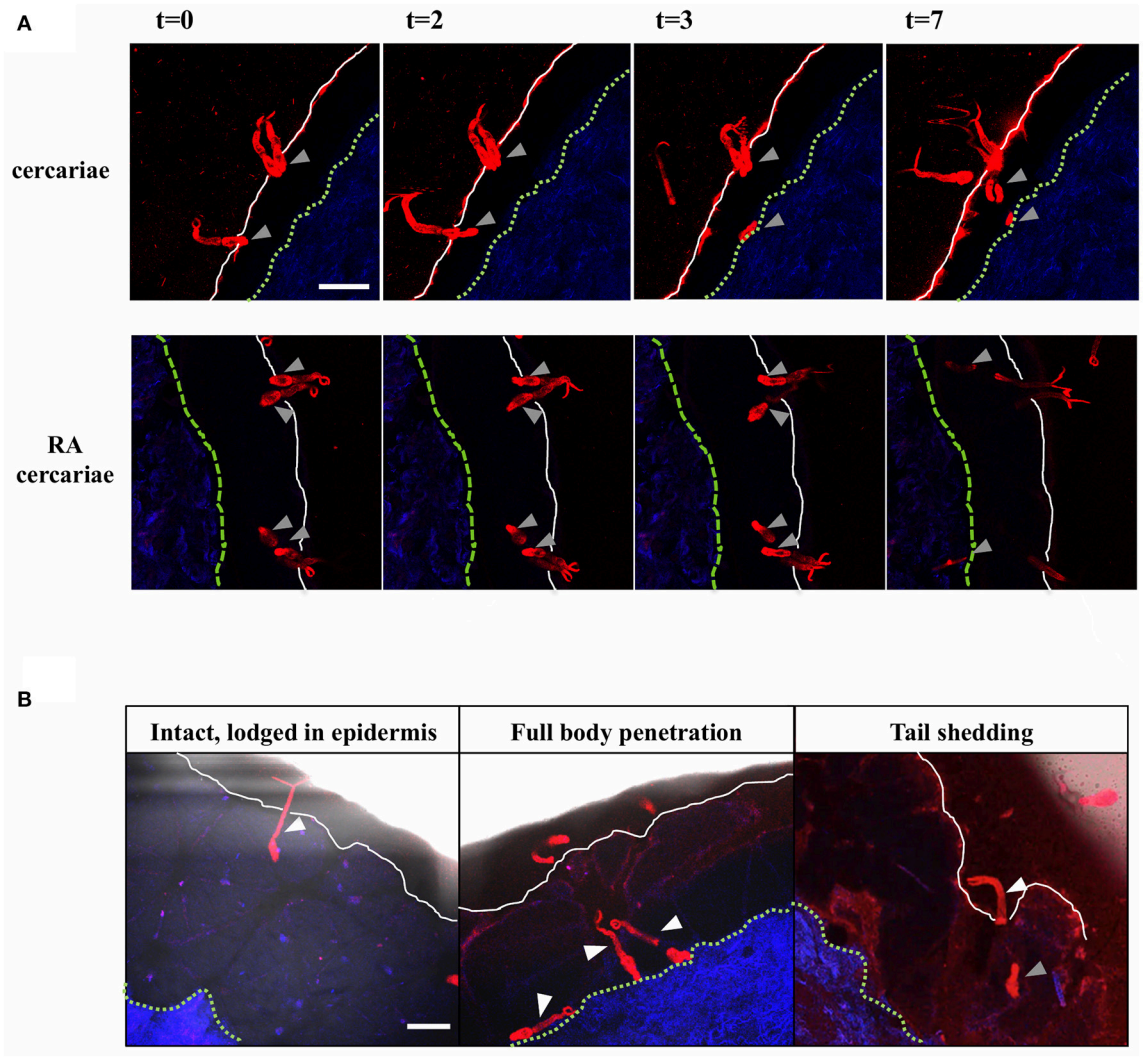
Visualization of cercarial invasion into a human skin explant. Cercariae attach and penetrate the epidermis of the human skin explant. Cercariae depicted in red, the dermis in blue, the epidermal surface as a white solid line and the basal membrane as a green dashed line T = 0–7 min. Gray arrowheads: cercarial heads/schistosomula. Top panels: non-attenuated cercariae, lower panels: RA cercariae **(A)**. Cercariae penetrate the skin in different ways. White arrowheads: cercariae/tails (**B**). Scale bar: 200 μm.

Using our fluorescence-based *S. mansoni* cercariae imaging technique, we were able to monitor the invasion in human skin in real-time (Figure 2; Supplementary Videos 1, 2). The infectious potential of non-attenuated as well as RA cercariae was confirmed by microscopically imaging the skin interaction. Larvae were seen to attach to the epidermal surface with their tails thrashing, after which they invaded deeper into the epidermis with gliding motion. Twenty-three of the forty-five non-attenuated and 18 of the 35 RA cercariae studied (51%) entered the human skin within 30 min (non-attenuated cercariae median: 8.5 min, range: 5–10 min Inter Quartile Range (IQR) 2.13 min; RA cercariae median 7.5 min, range 6.5–30 min, IQR 1.5 min) (Figure 2A; Table 1). This invasion time roughly corresponds with previous data in which the authors self-infected with RA cercariae [mean penetration time 6.58 min, 1.57–13.13 min (45)]. In general we recorded three different ways of invasion; (1) cercariae penetrated with their heads, remain intact and stay lodged in the epidermis, (2) intact cercariae penetrated the full thickness of the epidermis, and (3) cercariae penetrated with their heads, shedding their tail on the surface, continuing on as schistosomula (Figure 2B; Table 1). These different ways of invasion were seen for both non-attenuated as well as RA cercariae, although RA cercaria seemed to shed their tail more readily. It is interesting to note that all cercariae which penetrated the epidermis halted migration at the epidermal-dermal junction (time frame 30 min).

**Table 1:** Skin invasion by cercariae.

	**Cercariae**	**RA Cercariae**
No of imaged cercariae	45	35
Invaded	23 (51%)	18 (51%)
Lodged in epidermis	4	5
Intact penetration	14	4
Tail shedding	5	9

### Non-attenuated cercariae induce regulatory dermal immune responses

**Figure 3:**
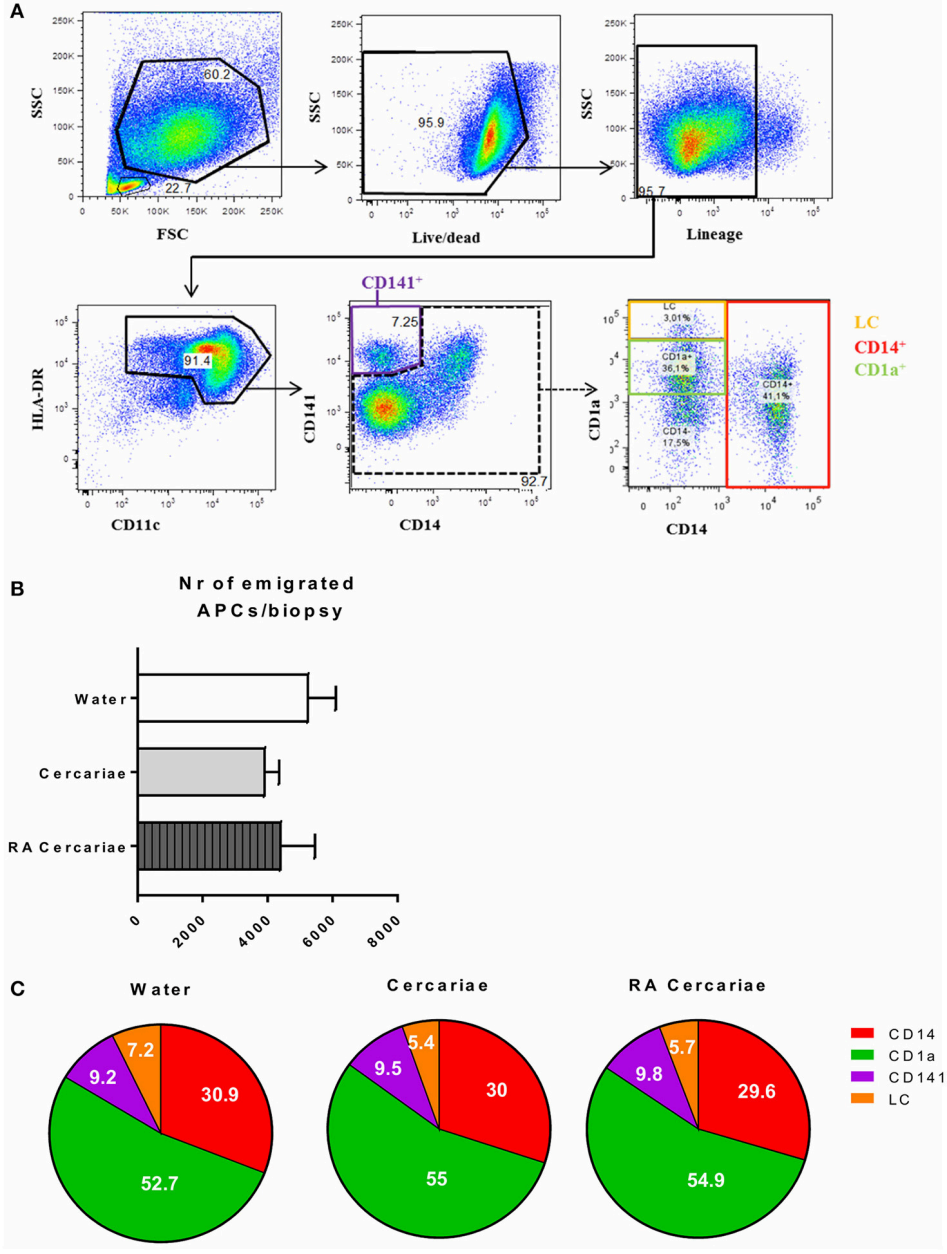
*S. mansoni* cercariae do not induce DDC emigration from the skin. DDC gating strategy. Antigen presenting cells are selected by forward scatter (FSC) and side scatter (SSC) characteristics. Doublets are excluded (not shown). Live cells are gated and selected for the lack of lineage markers (CD56, CD3, CD19, CD20). APCs are selected on the expression of HLA-DR as well as intermediate to high levels of CD11c. HLA-DR^+^, CD11c^+^ cells can be divided into the different DDC populations: CD141^+^, LC, CD1a^+^ and CD14^+^ **(A)**. Total emigrated HLA-DR^+^, CD11c^+^ antigen presenting cells from dermal biopsies at 3 days post exposure to *S. mansoni* cercariae or water control. Mean ± SEM, *n* = 7 (**B**). Subset distribution of emigrated cells (**C**).

**Figure 4:**
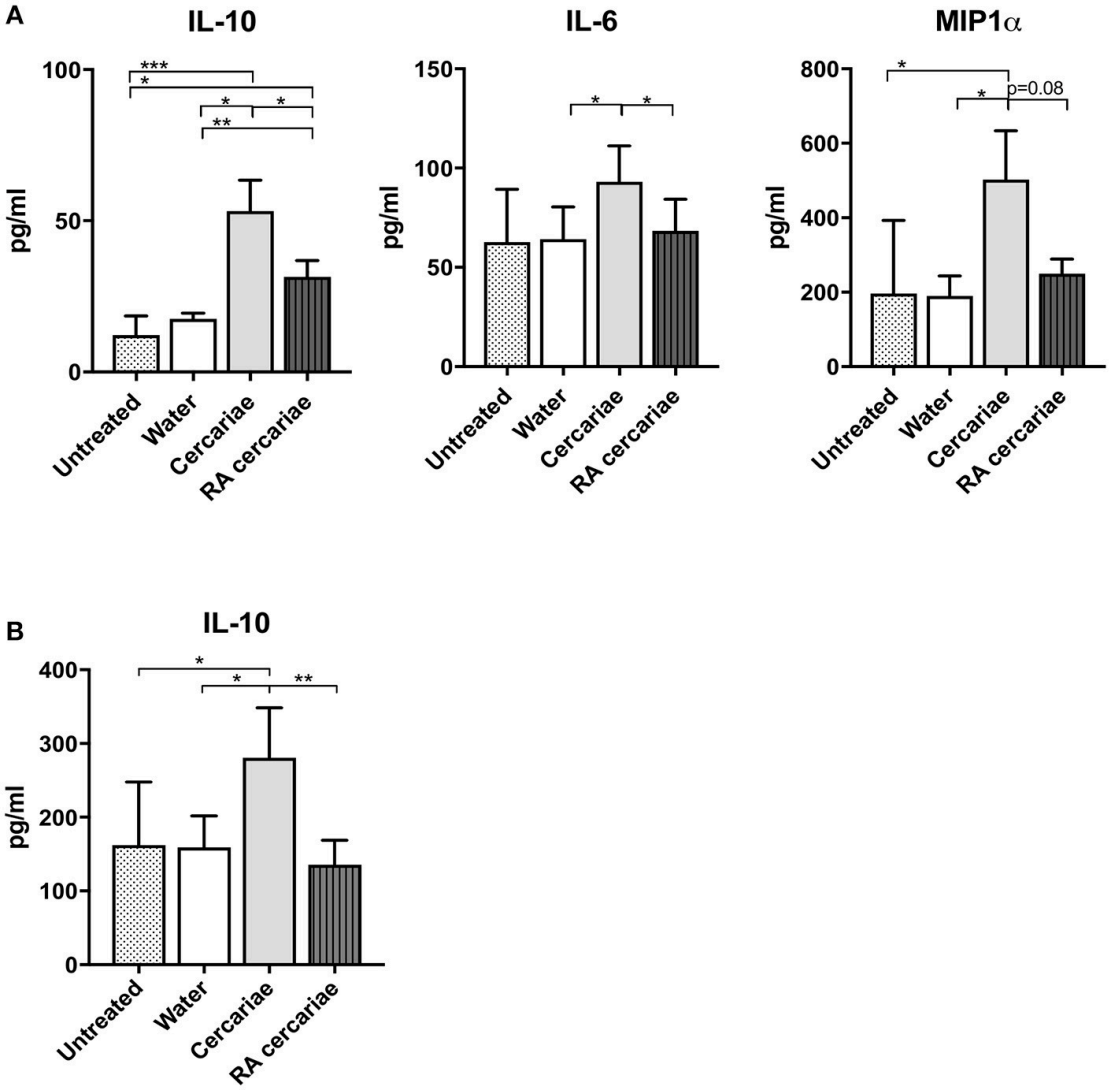
Increased production of IL-6 and IL-10 and MIP1α in skin exposed to cercariae. Whole biopsy cytokine analysis at 3 days post exposure to non-attenuated Sm cercariae show an increase in IL-6, IL-10 and MIP1α. The effect is less pronounced in radiation attenuated cercariae. **(A)**. IL-10 production by DDCs after co-culture of emigrated DDCs with CD40L expressing cell line, 7 donors. (**B**). Data shown in pg/ml, mean ± SEM. ^*^p < 0.05, ^**^p < 0.01, ^***^p < 0.001 using paired Student's *T-*test on log transformed data.

To start addressing the innate immunological responses to cercarial exposure in *ex vivo* exposed human skin biopsies, we determined the subset distribution of the various crawl-out DC populations. Neither exposure of human skin to *S. mansoni* non-attenuated or RA cercariae induced migration of skin APCs or altered their subset distribution (Figure 3; Supplementary Figure 1). However, analysis of the whole biopsy cytokine environment exposed to non-attenuated cercariae revealed an average 2.7-fold increase in the regulatory cytokine IL-10, a 1.4-fold increase in the pro-inflammatory cytokine IL6 and a 3.7-fold increase in inflammatory chemokine of the innate immune response, macrophage inflammatory protein (MIP)1α (Figure 4A) compared to water exposed controls. These cytokine responses were less pronounced (1.7-fold for IL-10 and 1.6-fold for MIP1 α) or absent (IL-6) in tissue exposed to RA cercariae (Figure 4A). Additionally, we measured chemokines and cytokines previously reported in murine dermal *S. mansoni* models (MIP-1β, IL-4, IL-12p40, IL-18, IL-23, and IFNγ, (7, 11, 12, 46). However, IL-4, IL-12p40, IL-18, IL-23, and IFNγ were not detectable in our model. A trend similar to MIP-1α was seen for MIP-1β (data not shown). Next we studied whether skin APCs could be the source of IL-10 by co-culturing crawl-out DDCs with a CD40L–expressing B cell myeloma line, mimicking T cell interaction. We found that DDCs increased their IL-10 production upon exposure to non-attenuated cercariae, which was not seen in DDCs exposed to RA cercariae (Figure 4B).

**Figure 5:**
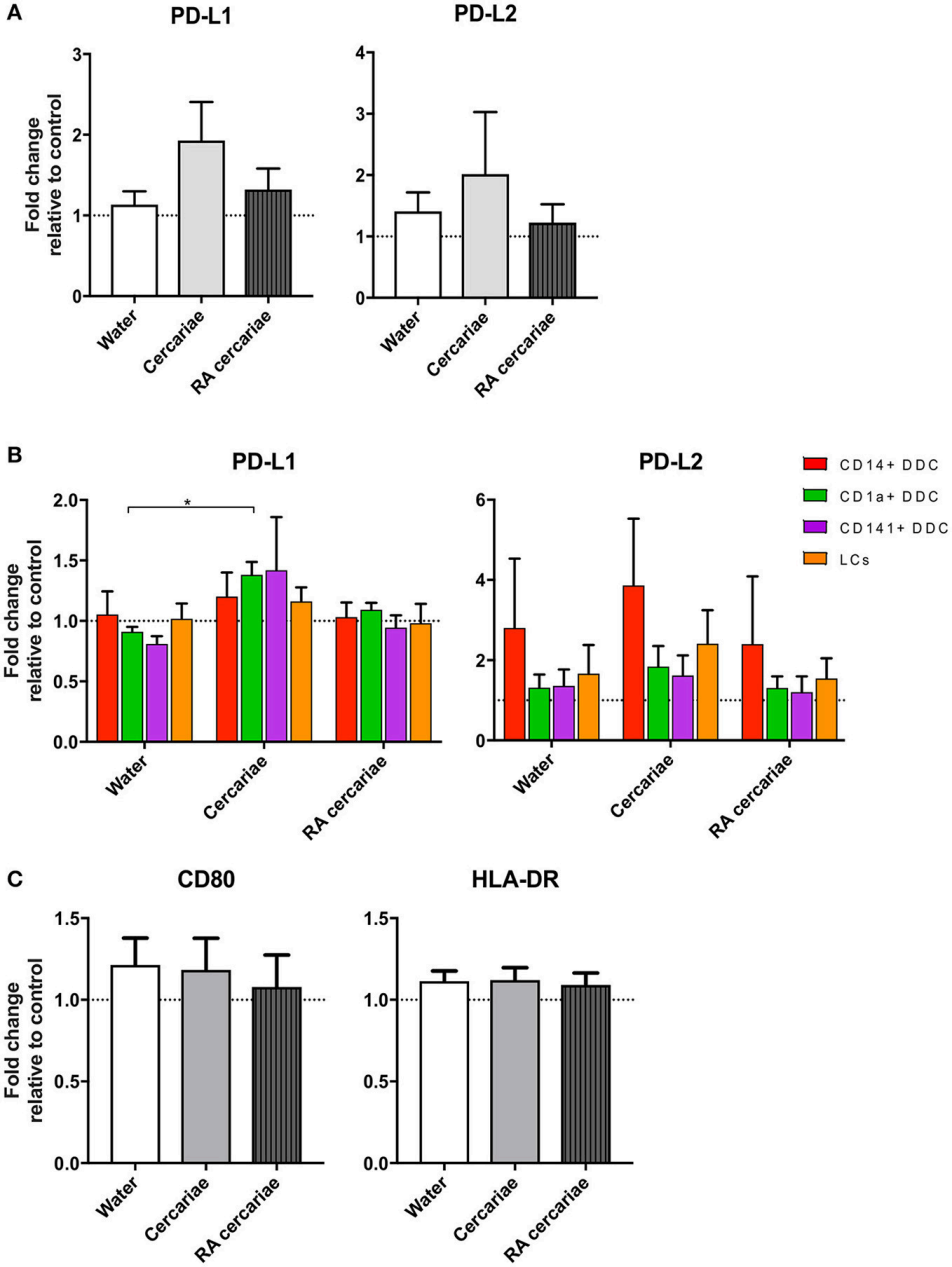
DDCs do not increase activation markers upon cercaria exposure but do show a trend of increased levels of immunoregulatory markers PD-L1 and PD-L2. Immunoregulatory markers PD-L1 and 2 are up regulated in DDCs when exposed to Cercariae but not RA Cercariae (*n* = 3) **(A)**. PD-L1 expression primarily occurs in CD1a^+^ DDCs. (*n* = 3) **(B)**. CD80 and HLA-DR expression in total emigrated HLA-DR^+^, CD11c^+^ antigen presenting cells from dermal biopsy at 84 h post exposure to *S. mansoni* cercariae or water control (*n* = 7) **(C)**. Mean ± SEM. ^*^p < 0.05.

We continued to investigate the phenotype of the exposed crawl-out DDCs in more detail and found an increased expression of immune-modulatory molecules programmed death ligand (PD-L) 1 and 2 (1.9- and 2-fold, respectively; Figure 5A) after exposure to non-attenuated cercariae. PD-L1 was primarily upregulated in the CD1a+ DDC population, whereas PD-L2 was overall more prominent in the CD14+ subset and in Langerhans cells (LCs; Figure 5B). For RA cercariae, this upregulation was less pronounced (1.3-fold increase for PD-L1) or absent (PD-L2; Figure 5A). Crawl-out DDCs did not up regulate activation markers CD80 and HLA-DR after (RA) cercariae exposure (Figure 5C).

**Figure 6:**
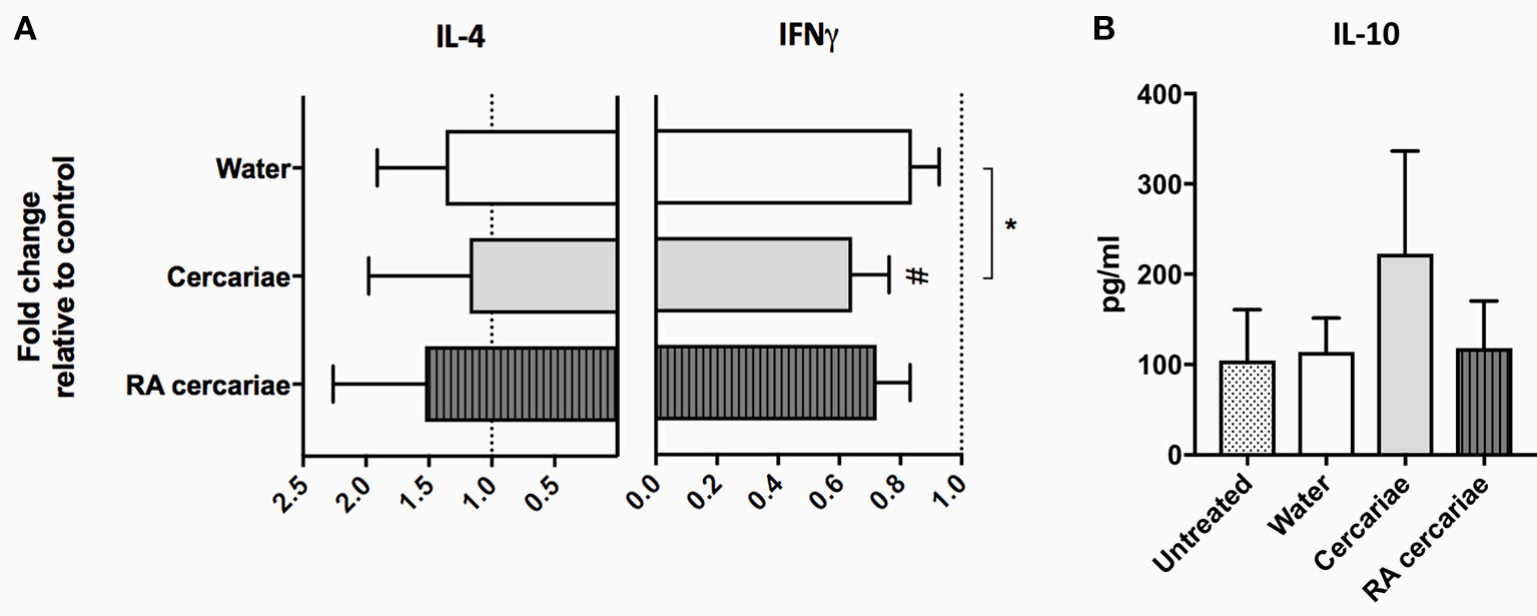
Stimulation with cercariae reduces pro-inflammatory potential of DDCs. Emigrated DDCs were co-cultured with CD4^+^ T cells to assess immunomodulatory potential. Data shown as percentages IFNγ or IL4 producing CD4^+^ T cells cultured with *ex vivo* stimulated DDCs relative to CD4^+^ T cells cultured with DDCs from untreated skin after PMA/ionomycin stimulation. Mean ± SEM. ^*^p < 0.05, using paired Student's *T*-test **(A)**. IL-10 production by CD4^+^ T cells after co-culture with DDCs. ELISA on culture supernatants after 24 h stimulation with anti CD3/28 **(B)**. Data shown in pg/ml. Mean ± SEM.

To assess the ensuing T cell responses we performed a co-culture assay of allogeneic naïve CD4^+^ T cells with (RA) cercariae exposed craw-lout DDCs. After co-culture with DDCs exposed to non-attenuated cercariae, CD4^+^ T cells produced less IFNγ and showed a trend of increased IL-10 production (Figure 6), suggesting regulatory potential of these DDCs. In line with our phenotypic analysis, this functional regulatory potential was not seen for RA cercariae exposed DDCs (Figure 6). A summary of the detected dermal immune responses can be found in Table 2.

**Table 2:** Immune phenotype of (RA) cercariae stimulated DDCs.

	**Cercariae**	**RA Cercariae**
**DDC Phenotype**
CD80	=	=
HLA-DR	=	=
PD-L1	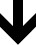	=
PD-L2	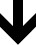	=
IL-10 production	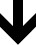 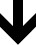	=
**Dermal Cytokines**
IL-10	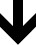 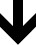	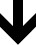
IL-6	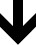	=
MIP 1	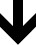 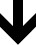	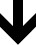
**NaÏVE T-Cell Responses to DDCs**
IFNγ	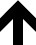	=
IL-4	=	=
IL-10	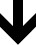	=

*The symbols =, 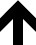, and 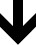 signify similar levels, a decrease or an increase, respectively, compared to water exposed control*.

### Direct contact with (RA) cercariae is necessary to induce a regulatory phenotype in monocyte-derived dendritic cells

**Figure 7:**
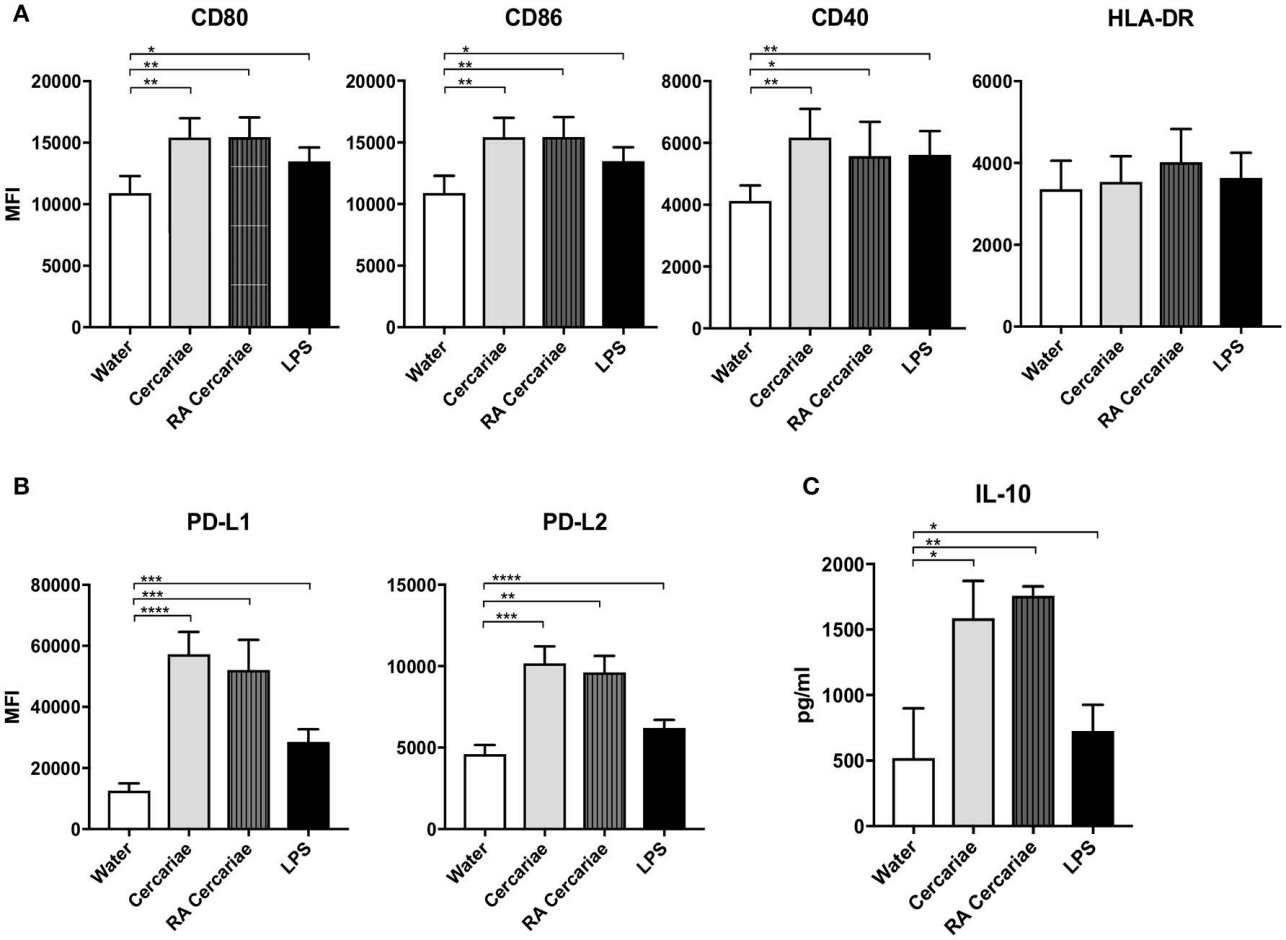
Cercaria stimulation activates MoDCs but also induces up regulation of immunoregulatory markers PD-L1 and 2 and regulatory cytokine IL-10 production. Stimulation of MoDCs with (RA) cercariae up regulates activation markers CD80, CD86 and CD40. **(A)**. PD-L1 and PD-L2 up regulation after cercaria stimulation. **(B)** IL-10 production by MoDCs after 48 h stimulation, data shown as pg/ml **(C)** Mean ± SEM. ^*^p < 0.05, ^**^p < 0.005, ^***^P < 0.0005, ^****^p < 0.0001 using paired student's *T*-test (on log transformed data for IL-10).

To further investigate APC phenotype in response to *S. mansoni* antigens, we generated monocyte-derived dendritic cells (moDCs) isolated from healthy volunteers and incubated these cells with (RA) cercariae. We found increased expression of activation markers CD80, CD86, and CD40, but not HLA-DR, (Figure 7A) in stimulated MoDCs. However, similar to their DDC counterparts, MoDCs strongly up regulated expression of immune regulatory molecules PD-L1 and PD-L2 (Figure 7B). Complementing these findings, MoDCs give an increased production of IL-10 upon stimulation with (RA) cercariae (Figure 7C). In contrast to exposure in skin, there was no significant difference between non-attenuated and RA cercariae in this *in vitro* setup. Table 3 summarizes the MoDC phenotype findings.

**Table 3:** Immune phenotype of (RA) cercariae stimulated Monocyte derived DCs.

	**Cercariae**	**RA Cercariae**
**MoDC Phenotype**
CD80	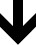	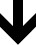
CD86	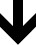	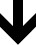
CD40	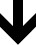	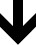
HLA-DR	=	=
PD-L1	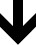 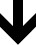	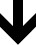 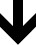
PD-L2	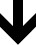 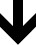	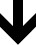 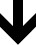
IL-10 production	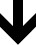 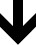	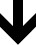 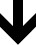

*The symbols =, 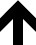, and 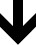 signify similar levels, a decrease or an increase, respectively, compared to water exposed control*.

**Figure 8:**
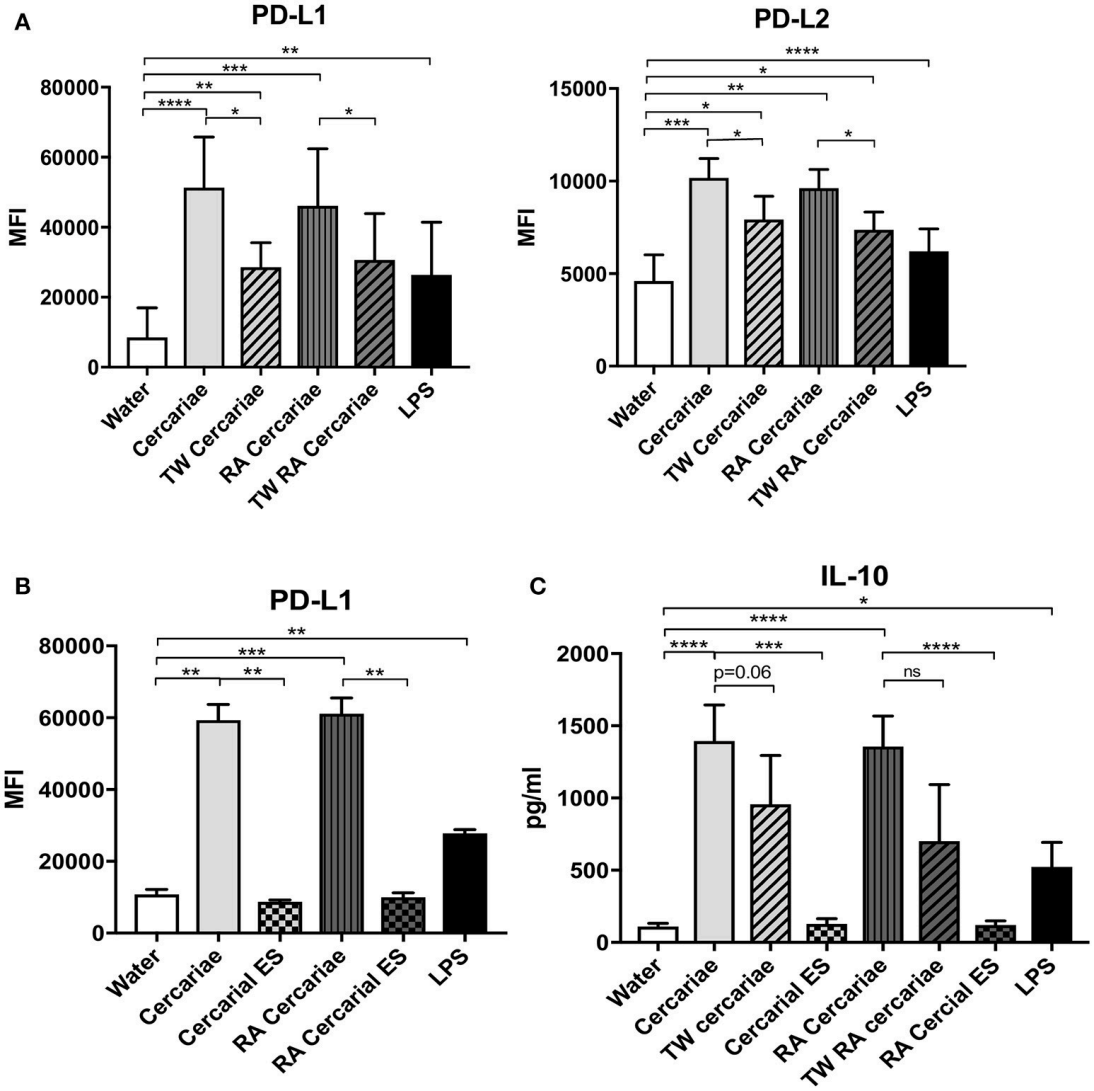
Direct contact with cercariae but not cercarial ES products increases immunoregulatory markers PD-L1 and 2 and IL-10 secretion. PD-L1 and PD-L2 up regulation after cercaria stimulation in presence or absence of direct contact (TW = transwell) **(A)**. PD-L1 expression after stimulation with cercariae or cercarial ES alone. The ES dose was matched to the number of cercariae used for stimulation (**B**). IL-10 production of MoDCs after stimulation with cercariae or their products **(C)**. Mean ± SEM. ^*^p < 0.05, ^**^p < 0.005, ^***^P < 0.0005, ^****^p < 0.0001 using paired student's *T*-test (on log transformed data for IL-10).

In order to dissect whether the immunosuppressive phenotype of DCs was induced by direct interaction of the APC with *S. mansoni* or a response to their ES products, we performed a transwell assay which physically separates the MoDCs from the (RA) cercariae. Interestingly, PD-L1 and PD-L2 upregulation was partly dependent on the direct contact of cells with (RA) cercariae (Figure 8A). And indeed, incubation of MoDCs with ES products of transformed (RA) cercariae alone did not induce PD-L1 and PD-L2 expression (Figure 8B). In addition to PD-L1 and PD-L2 up regulation, MoDCs increased their production of IL-10 only when allowed direct contact with (RA) cercariae, whereas transwell stimulation or ES stimulation did not induce IL-10 production (Figure 8C).

## Discussion

For the first time we visualized human skin invasion by *S. mansoni* cercariae and show that these larvae subsequently drive expression of PD-L1 and 2 and IL-10 by human DDCs, indicative of a regulatory phenotype. In line with their regulatory phenotype, these DDCs suppress Th1 priming, and favor the induction of regulatory cytokine IL-10 by T cells. RA cercariae were less capable of inducing skin immune suppression. These findings may help to explain why, in contrast to RA cercariae, exposure to non-attenuated cercariae fails to initiate a protective immune response. The early differences in the immune responses to non-attenuated and RA cercariae imply that priming of the protective immune response against RA *S. mansoni*, is initiated immediately after invasion, by APCs of the dermis. Whether immune priming in the skin draining lymph node alone is sufficient to establish protective immunity remains a long-standing matter of debate (47–49).

Previously published epidemiological data revealed that individuals infected with parasitic worms (helminths) such as *S. mansoni* show elevated serum levels of IL-10 (50, 51, 52, 53). Taken together, our results support the hypothesis that active suppression of immune responses is initiated immediately after infection, at the dermal stages of *S. mansoni* infection. This propensity of *S. mansoni* larvae to promote T-cell hyporesponsiveness may, in part, explain why cercarial dermatitis is a subtle clinical phenomenon (10, 18).

The human skin cytokine responses to *S. mansoni* correspond with data in murine models, which also show a dermal invasion site rich in IL-10 and a less pronounced increase in pro-inflammatory cytokines IL6 and MIP-α and β (7, 11, 12, 46). The source of IL-10 in the skin is still unclear. Several dermal cell-types, such as lymphocytes and keratinocytes have been shown to produce IL-10 in response to *S. mansoni* larvae (18, 54). Additionally, dermal mast cells are a known source of IL-10 in a variety of skin diseases (54) and cercarial ES products have been shown to affect these cells by triggering their degranulation. Our data, however, suggests that dermal APCs are an important source of dermal IL-10 enrichment in response to cercariae. In addition, we observed a small increase in the levels of pro-inflammatory cytokine IL-6 and innate inflammatory chemokine MIP-1α. These results are in line with previous reports using murine dermal *S. mansoni* models (7) and highlight that the cumulative dermal immune response to *S. mansoni* is a delicate balance between innate pro-inflammatory signals and a modulatory IL-10 response. In RA cercariae, this immune suppression is less pronounced, corroborating murine skin data (7). Also in line with murine data (46), our research shows increased IL-10 production by naïve CD4^+^ T cells after co-culture with cercariae-exposed DDCs. However, contrary to what has previously been found in the murine skin draining lymph node (55) we detected a significant reduction in Th1 priming of CD4^+^ T cells in response to non-attenuated cercariae, highlighting differences between human and mouse skin responses in *S. mansoni* infection. Despite the fact that the *ex vivo* human skin explant model lacks blood flow, excellent viability of dermal cells in cultured skin biopsies is maintained for a long period of time (56, 57), making this model a valuable method which can aid in understanding human immune responses during natural infections in a three dimensional organization.

A potential mechanism by which *S. mansoni* is able to regulate immune activation is the excretion of ES products, which have been described to exhibit regulatory potential (19, 58–60). However, we find that direct contact of the parasite with immune cells is needed to induce the immunosuppressive effects on human MoDCs. Interestingly, immune-modulatory markers PD-L1 and 2 were primarily induced by non-attenuated cercariae in the skin explant model, while *in vitro* MoDCs up regulated these markers in response to direct contact with both non-attenuated as well as RA cercariae. This may indicate that dermal migratory behavior of (RA) cercariae may influence DDC-cercarial contact and thus alter the ensuing immune responses as has been previously suggested (5, 38). Because migration does not affect the cellular contacts in the *in vitro* assay, this effect may be lost *in vitro*. Using a cell-labeling dye we visualized *S. mansoni* cercarial penetration in human skin and confirmed cercarial penetration. Using this technique we could clearly visualize the fate of the tail in the skin. A previous study applying confocal imaging to investigate cercarial invasion into mouse ear pinnae showed comparable invasion behavior, although all cercariae imaged in this study displayed tail loss upon entry (61). Tail loss at the moment of skin entry has been assumed previously, but contradicted by some authors (45, 62, 63). It has been suggested that delayed tail loss might be a mechanism by which the host immune response is diverged away from targeting adult worms (62). RA cercaria seem to be less capable of doing so. Potentially, differences in the timing of tail shedding between non-attenuated and RA cercariae may be responsible for the differential immunological effect in skin. After penetration, *S. mansoni* cercariae shed their surrounding glycocalyx, which protects them during the aquatic stage of the life cycle. This shedding exposes the cercarial membrane and its molecules and therefore timing may be critical to the ensuing immune response. Although phagocytosis of cercarial ES products by APCs in the dermis results in their activation and the production of pro-inflammatory cytokines (61), surprisingly little is known about membrane-bound molecules on cercariae and/or schistosomula and the DDC receptors that recognize them during direct contact. Further research is needed to fully understand the molecular basis through which *S. mansoni* modifies APC function at this stage of the life cycle.

Our finding that *S. mansoni* cercariae induce upregulation of PD-L1/2 in both DDCs as well as MoDCs suggests that, similar to cancer cells, *S. mansoni* may exploit the PD-1 pathway to inhibit the adaptive immune response starting in the human dermis. Recently, PD-L1 and PD-L2 upregulation has been demonstrated in monocytes exposed to *Brugia malayi*, the causative parasitic agent for filariasis (64), indicating that the effect on PD-L1/2 in phagocytic APCs may be part of an immune-regulatory pathway employed by different parasites. This suggests PD-1 or PD-L1/2 targets could potentially be used in the development of vaccines against parasitic diseases.

In conclusion, we visualized cercarial invasion into human skin and demonstrate that, similarly to rodent models, *S. mansoni* cercariae are able to induce a regulatory dermal immune response. In our human model, this response is characterized by expression of PD-L1/2, excretion of IL-10 and the suppression of IFNγ producing CD4^+^ T cells. This process is less well mastered by RA cercariae, which may explain, in part, why they are superior immunogens. An understanding of the immune suppressive capacity of *S. mansoni* in human skin may give clues toward the development of novel therapies, or directly impact the development of an effective schistosome vaccine.

## Ethics statement

The use of human skin explants (obtained as waste material after abdominal reduction surgery) for this research was approved by the Commission Medical Ethics (CME) of the LUMC, Leiden. Approval number CME: B18-009.

## Author contributions

The methodology was developed by BW, CdK, FvL, BE, LP, MY, EdJ, and MR. Experiments were performed and interpreted by BW, MD, CF, ML, CdK, MG, and LP and supervised by HS, CH, BE, and MR. BW and MR drafted the manuscript. All authors reviewed and contributed to finalizing the manuscript.

### Conflict of interest statement

The authors declare that the research was conducted in the absence of any commercial or financial relationships that could be construed as a potential conflict of interest.
