# Supplementary figures and images for: Early Induction of Human Regulatory Dermal Antigen Presenting Cells by Skin-Penetrating Schistosoma Mansoni Cercariae
Source: Front Immunol. 2018 Oct 31;9:2510. doi: 10.3389/fimmu.2018.02510 (PMC6220649; doi:10.3389/fimmu.2018.02510)

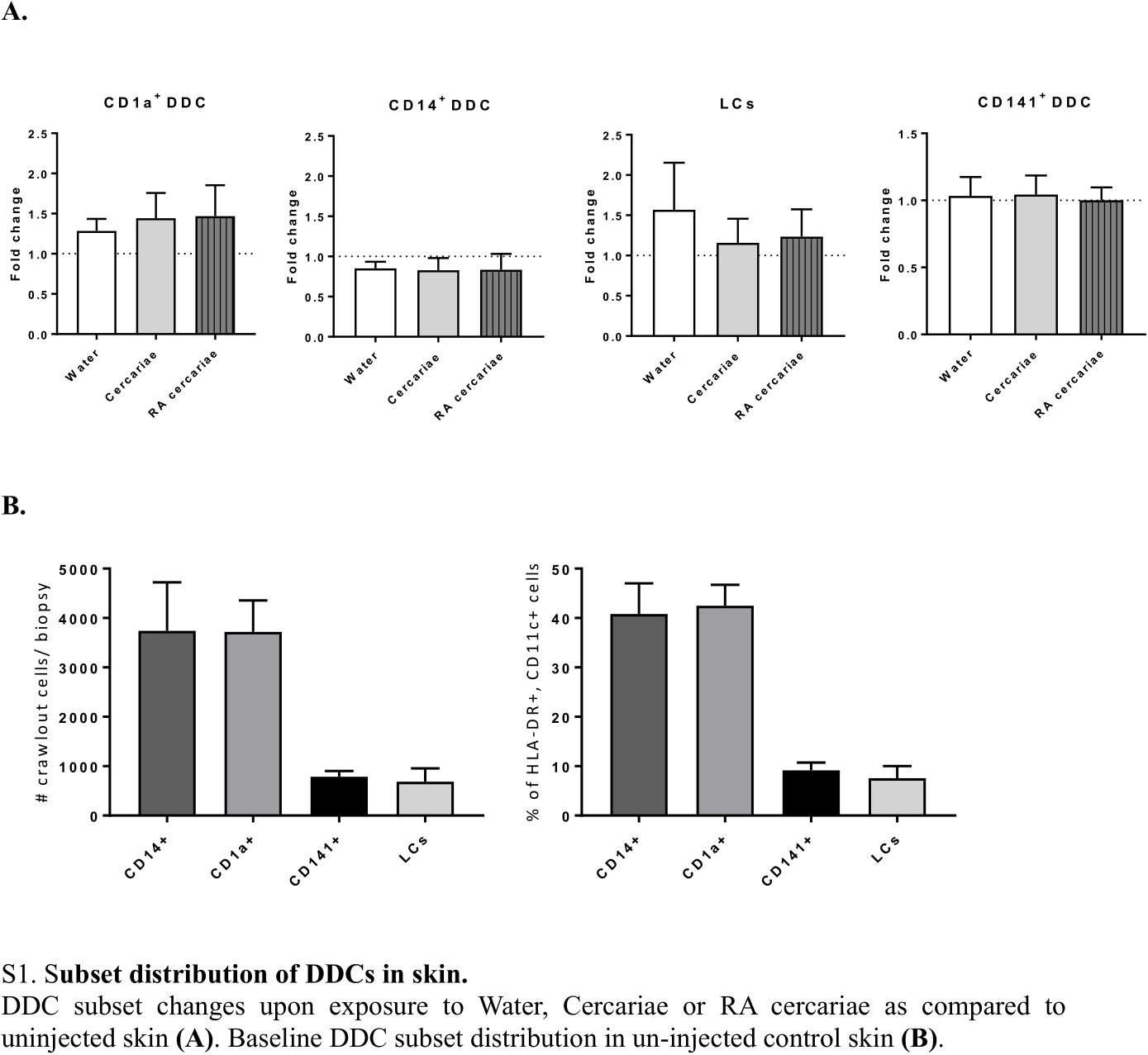

Supplement: Supplementary file 3 [file Image_1.tiff]
